# Hydrogen peroxide treatment induces the transposition of an insertion sequence in *Deinococcus radiopugnans* DY59

**DOI:** 10.3389/fmicb.2023.1110084

**Published:** 2023-03-02

**Authors:** Eunjung Shin, Hee Seong Noh, Qianying Ye, Sung-Jae Lee

**Affiliations:** Department of Biology, Kyung Hee University, Seoul, Republic of Korea

**Keywords:** *Deinococcus radiopugnans* DY59, genome plasticity, insertion sequences, oxidative stress, phenotypic selection, transposition

## Abstract

*Deinococcus radiopugnans* DY59 (formerly *Deinococcus swuensis* DY59) is a radiation-resistant bacterium isolated from soil. From the 3.5 Mb genomic DNA sequence of strain DY59 (December 2014), 31 insertion sequence (IS) elements of six IS families including IS*1*, IS*4*, IS*5*, IS*66*, IS*630*, and IS*701* and five unclassified IS elements were detected. Upon induction of oxidative stress with 80 and 100 mM H_2_O_2_, the unique ISs of the IS*4* family member were actively translocated into a carotenoid biosynthesis gene phytoene desaturase (QR90_10400), resulting in non-pigment phenotypic selection. Therefore, these active transpositions of a specific IS family member were induced by oxidative stress at 80 and 100 mM H_2_O_2_. Furthermore, *D. radiopugnans* DY59 exhibited extremely higher MIC values against H_2_O_2_ treatment. To explain this phenomenon, qRT-PCR was conducted to assess the expression levels of catalase and three LysR family regulators. Our findings indicated that the IS*Drpg2* and IS*Drpg3* elements of the IS*4* family were actively transposed into the phytoene desaturase gene by H_2_O_2_ treatment *via* replicative transposition. However, high H_2_O_2_ resistance did not originate from H_2_O_2_-induced expression of catalase and LysR family regulators.

## Introduction

Most species belonging to the genus *Deinococcus* exhibit extreme resistance to gamma irradiation, ultraviolet ray exposure, and desiccation, which makes them ideal model microorganisms for studying anti-oxidation mechanisms. Particularly, these models allow for the assessment of the specific DNA repair and redox-sensing regulation mechanisms of cells based on the functional roles of genus-specific proteins for DNA damage responses (Daly, 2009; Slade and Radman, 2011; Luan et al., 2014; Lim et al., 2019).

*Deinococcus swuensis* DY59^T^ was isolated by separating gamma-ray-resistant microorganisms from a soil sample obtained from the Deogyusan mountain (Jeonbuk Province, South Korea) at 1,500 m altitude (Lee et al., 2013). The soil was irradiated with 5 kGy gamma rays using a cobalt-60 gamma irradiator. Strain DY59 exhibited survival rates of 21 and 1% when exposed to 5 and 10 kGy of gamma radiation, respectively (Lee et al., 2013). The DY59 cells were found to be aerobic, coccus-shaped, had little mobility, and formed pink colonies in Luria-Bertani agar plates. The published genome of DY59 consisted of a single chromosome of 3,531,443 bp with a G + C content of 67.4%, which included 3,305 protein-encoding genes and 58 RNA genes (GenBank accession number GCA_000800395.1, December 2014; Kim et al., 2015). Upon sequencing of the 16S rRNA gene, strain DY59^T^ showed high sequence similarity (99%) with the *Deinococcus radiopugnans* type strain (ATCC19172) as well as to *Deinococcus marmoris* KACC12218^T^ (97.9%), *Deinococcus saxicola* KACC12240^T^ (97.0%), *Deinococcus aerolatus* KACC12745^T^ (96.2%), and *Deinococcus frigens* KACC12220^T^ (96.1%; Lee et al., 2013; Kim et al., 2015). Recently, *D. swuensis* DY59 was reclassified as *Deinococcus radiopugnans* following genome data analysis including average nucleotide and amino acid identity and digital DNA–DNA hybridization on July 2021 (Lakra et al., 2021).

Prokaryotic genomes exhibit the smallest transposable element insertion sequences (ISs). They have important roles in genomic evolution including the enhancement of gene inactivation and genome plasticity (Touchon and Rocha, 2007; Siguier et al., 2014, 2015). IS abundance is positively correlated with the frequency of horizontal gene transfer (HGT), genome size, pathogenicity, non-obligatory ecological associations, and human association (Touchon and Rocha, 2007; Vandecraen et al., 2017). Moreover, recent mobilome studies have characterized IS distribution at the genus level (Blesa et al., 2019; Fayad et al., 2019). IS elements are transferred between genomes through all classical mechanisms of HGT (Frost et al., 2005). IS elements generally consist of one or two transposase ORFs and a terminal inverted repeat (TIR) sequence. Transposases are the most prevalent genes in nature. When IS element integrated into genomic DNA, the direct repeat (DR) sequences were produced generally. Unique and random DR sequences in border of IS elements indicated a specific and random sequence recognition and integration. Their mobile nature not only promotes the dissemination of transposable elements within and between genomes but also leads to mutations and rearrangements that can accelerate biological diversification and consequent evolution (Aziz et al., 2010).

In the case of *Deinococcus geothermalis* (Dgeo), 73 full-length IS elements belonging to nine IS families were distributed across the different molecules of the genome including two mega plasmids. Several IS elements were actively transposed into other genome loci through the copy-and-paste mechanism of IS*Dge5*, IS*Dge6*, and IS*Dge7* members under hydrogen peroxide (H_2_O_2_)-induced oxidative stress. Furthermore, we reported the occurrence of a colorless phenotype through the loss of carotenoid biosynthesis *via* IS transposition, which was supported by several lines of evidence (Tian and Hua, 2010; Lee et al., 2019, 2020; Ye et al., 2021; Shin et al., 2022). Research on the insertion sequence of bacteria can be conducted from a molecular evolution perspective, including cutting-edge mutagenesis using transposable elements to examine the plasticity of bacterial genomes induced by long-term culture or oxidative stress. It can also serve as a basic study of genome stability for the potential application of bioremediation in the genus *Deinococcus* species (Gerber et al., 2015; Wright et al., 2017; Consuegra et al., 2021).

In this work, we describe that the genome of *D. radiopugnans* DY59 has a total of 36 IS elements with six IS families including five unclassified IS elements. Interestingly, strain DY59_IS elements have multiple copies of the IS*66*, IS*630*, IS*701*, IS*4*, and IS*5* families. Nevertheless, when strain DY59 was exposed to 5 kGy of gamma irradiation, non-pigmented mutants were not observed (Ye et al., 2022). Here, we evaluated the occurrence of active transposition induced by short-term oxidative stress conditions and its effects on the genomic plasticity in the radiation-resistant bacterium *D. radiopugnans* DY59. When oxidative stress was induced *via* hydrogen peroxide treatment with 80 mM, the IS*Drpg2* and IS*Drpg3* IS elements of the IS*4* family were transposed into a carotenoid synthesis gene encoding a phytoene dehydrogenase (QR90_10400), resulting in a non-pigment phenotype. Furthermore, strain DY59 exhibited a high minimum inhibitory concentration (MIC) of 300 mM H_2_O_2_. Thus, the expression levels of catalase and LysR family members (i.e., its putative controlling regulators) were determined by qRT-PCR.

## Materials and methods

### Bacterial species and culture conditions

*Deinococcus radiopugnans* DY59 was kindly provided by Dr. M. Kim at Seoul Women’s University. This strain was deposited into the Korean Collection for Type Cultures (KCTC33033^T^). *Deinococcus geothermalis* DSM11300^T^ and *D. radiodurans* R1 were used as a positive control for the active transposition of ISs. *Deinococcus geothermalis* and *D. radiodurans* R1 were cultured on TGY medium containing 1% tryptone, 0.5% yeast extract, and 0.1% glucose with 150 rpm for broth culture at 48 and 30°C, respectively. Strain DY59 is known to grow well in R2A or LB medium. Therefore, the cells were cultured for 3–4 days at 30°C in R2A medium, whereas the cells in the TGY medium required only 2–3 days to grow. All downstream *Deinococcus* cultures were thus conducted using TGY culture medium.

### Selection and determination of active transposition

*Deinococcus radiopugnans* DY59 cells were typically grown to a maximum optical density (OD_600_) for 2–3 days. When the culture reached an absorbance of 2.0 or 4.0 at OD_600_ as measured by a UV–VIS spectrophotometer, the cells were harvested by centrifugation at 3,000 rpm and resuspended in a 0.9% NaCl solution to an OD_600_ of 2.0 for exposure to H_2_O_2_.

Next, the cells were treated with final H_2_O_2_ concentration ranges of 80 and 100 mM/200 and 300 or 0 mM as a control and continuously cultured for 1 h at 30°C with 150 rpm shaking. The samples were then directly diluted to 10^−4^–10^−5^ with 0.9% NaCl solution and 100 μL of the cell suspensions were spread on TGY agar medium using sterilized glass beads. After discarding the glass beads, the TGY plates were incubated at 30°C for 2–3 days. Non-pigmented colonies were detected and cell culture dilutions were streaked on TGY agar plates for pure cultivation. Non-pigment forming frequency was calculated the ratio of number of non-pigment colony and CFU. We also evaluated whether hypochlorite and gamma irradiation induced the occurrence of IS transposition. Active transposition was detected *via* PCR amplification of four selected target genes related to carotenoid biosynthesis (Supplementary Table S1).

### Detection of IS elements from genome sequence and non-pigment mutants

The *D. radiopugnans* DY59 genome sequence was obtained from the NCBI database (GenBank accession number NZ_CP010028.1). IS elements were detected from the annotated transposase genes using protein profiling data obtained from the GenBank database. We first extracted sequences from upstream and downstream of a transposase (1 kb region; 3 kb total length). Then, terminal inverted repeat (TIR) sequences and direct repeat (DR) sequences were determined using DNA sequence alignment software such as BLAST, ClustalW, and BioEdit. All detected IS elements were sorted into IS family members. Unfortunately, the IS detection platform ISfinder (https://isfinder.biotoul.fr; Siguier et al., 2006) still does not support the detection and classification of *D. radiopugnans* DY59 IS elements with calling ID as of September 2022. Therefore, we classified the IS ourselves based on IS structure analysis and nomenclature rules (Supplementary Table S2).

### Assessment of various stressors and MIC of antibiotics

Afterward, 10 ml of cultured cells till to an OD_600_ of 2.0 and 4.0 were harvested, resuspended with 0.9% NaCl, and separately exposed for 1 h to various H_2_O_2_ concentrations or 100 μg/mL sodium hypochlorite treatment or for in total 5 kGy gamma-irradiation (160 Gy/min). Then, to measure the cell viability using colony forming units, samples were diluted to a 10^−5^ ratio and spread on TGY agar plates. After 5 kGy gamma-irradiation, DY59 strain was exhibited 0.5% viability comparing to non-radiation. The viability of hypochlorite treatment was not analytical determined. To measure the minimum inhibitory concentration (MIC) of antibiotics, the strain DY59 cells of 100 μL on OD_600_ = 4.0 were spread on TGY agar plates, and the disk diffusion assay was conducted with different concentrations of 10 μL antibiotic solutions, such as streptomycin, kanamycin, and ampicillin. The MIC values were determined based on the formation of a clear zone.

### qRT-PCR analysis of catalase and three LysR family members

Strain DY59 cells were harvested at OD_600_ values of 2.0 and 4.0, resuspended to OD_600_ of 2.0 in 5 mL 0.9% NaCl, and then treated with 50–150 mM H_2_O_2_ for 1 h at 150 rpm. After the H_2_O_2_ challenge, the samples were centrifuged at 10,000 rpm for 5 min and washed once more with 0.9% NaCl. The supernatants were discarded and the pellets were stored at −20°C. The cell wall was broken using phenol and DNA digestion was performed using DNase I. RNA was extracted using an RNA prep kit for RNA isolation (RNeasy mini purification kit; Qiagen, Germany). After measuring the extracted RNA concentration, the concentration was normalized to 1,000 ng in an 8 μL volume for all samples. cDNA synthesis was performed with a dNTP mixture and 6-mer random primers using the following protocol: 60°C for 5 min, 4°C for 3 min, 30°C for 10 min, 42°C for 60 min, and finally 95°C for 5 min (PrimeScript™ first strand cDNA Synthesis Kit; TaKaRa, Japan). In the step at 4°C for 3 min, 4 μL 5× buffer, 4.5 μL RNase free water, 1 μL RTase, and 0.5 μL RNase inhibitor were added. qRT-PCR analysis was performed using TB Green® Premix Ex Taq™ (TaKaRa, Japan) on a Bio-RAD RT-PCR model CFX96™ Optics Module (Bio-RAD, United States). The expression of catalase (QR90_06310) and three LysR family regulators (QR90_13110, QR90_14595, and QR90_15105) was normalized to that of GAPDH, a gene that is constitutively expressed throughout all growth phases. The related expression levels of both catalase and LysR genes were calculated as described in a previous study (Choo et al., 2020). Differences between samples were determined *via* the Student’s *t*-test using the Prism™ software (ver. 8.0). Differences were deemed statistically significant at *p* < 0.05 (^*^) and *p* < 0.01 (^**^). The primer sequences for qRT-PCR were shown in Supplementary Table S3.

## Results

### Physiological properties and genetic distribution of carotenoid biosynthesis

**Figure 1:**
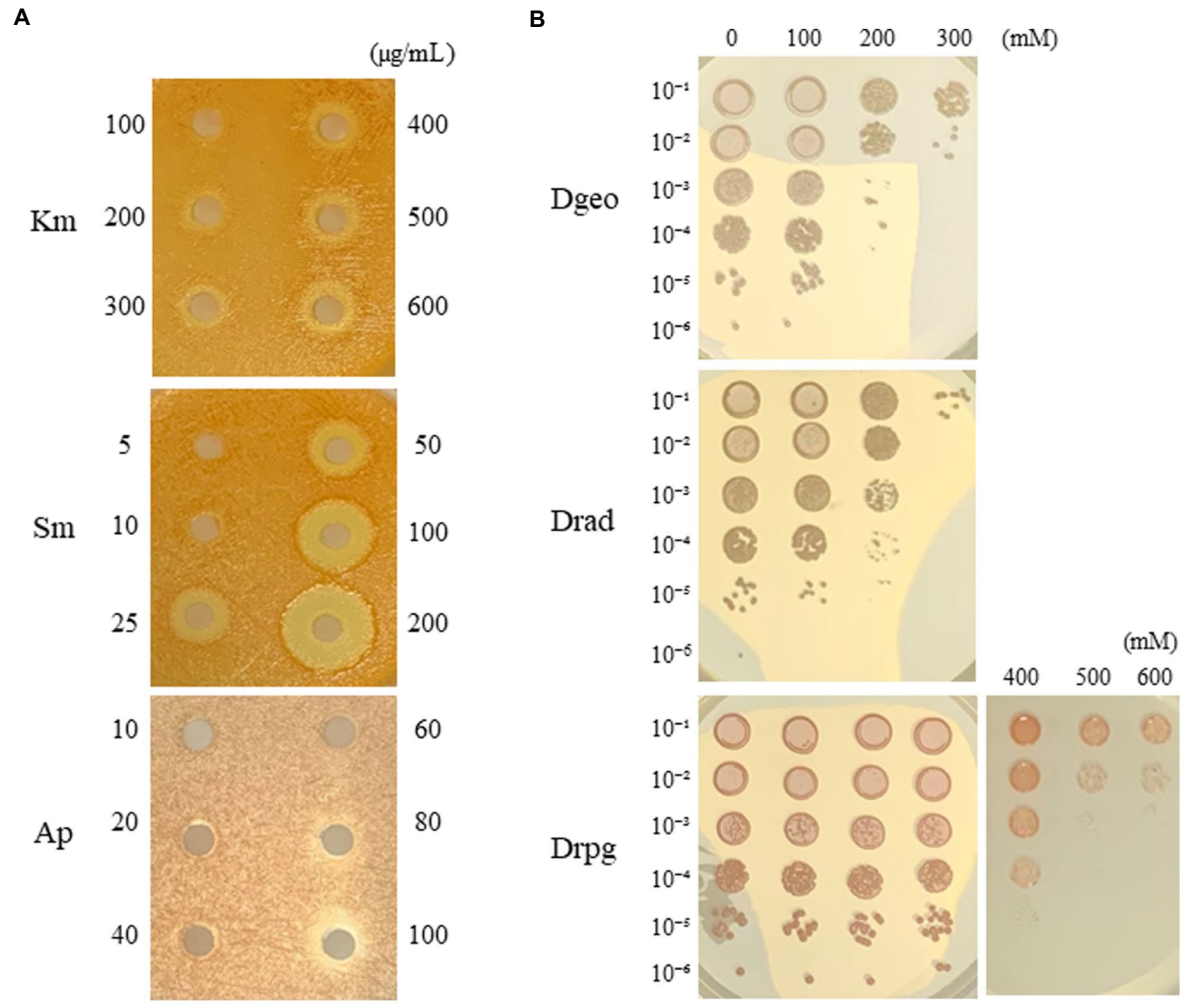
Minimum inhibitory concentration (MIC) measurement of kanamycin, streptomycin, and ampicillin *via* the disk diffusion method on *D. radiopugnans* DY59 **(A)** and viability assay under different concentrations of H_2_O_2_ among Drad, *D. radiodurans*; Dgeo, *D. geothermalis*; Drpg, *D. radiopugnans* **(B)**. Three *Deinococcus* species were grown to OD_600_ of 4.0, harvested, and resuspended by 0.9% NaCl to OD_600_ of 2.0. H_2_O_2_ treatment was performed on different final concentrations for 1 h then, serially diluted and spotted on TGY medium.

Generally, *D. radiopugnans* DY59 formed reddish-colored colonies after being cultured for 2–3 days on TGY medium at 30°C. The strain also exhibited a stronger viscosity compared to *D. geothermalis*. Here, we observed different MIC values, which were indicative of different levels of antibiotic resistance. The MIC of streptomycin was less than 25 μg/mL, whereas those of ampicillin, kanamycin, and chloramphenicol were 30, 300, and 60 μg/mlL, respectively (Figure 1A; Supplementary Figure S1). Therefore, we conclude that strain DY59 displays a resistant phenotype to kanamycin and chloramphenicol, and a tolerant phenotype to streptomycin and ampicillin when compared to the typical working concentrations of antibiotics.

Surprisingly, the results of our H_2_O_2_ viability assays indicated that *D. radiopugnans* DY59 could form colonies after being exposed to H_2_O_2_ concentrations of up to 600 mM for 1 h, which is two times higher than the resistance of two well-studied control strains of *D. radiodurans* and *D. geothermalis* (300 mM H_2_O_2_; Figure 1B).

**Figure 2:**
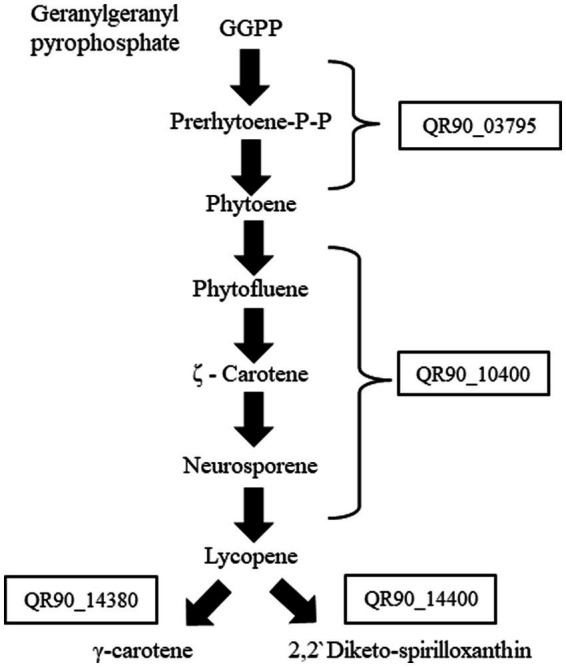
Simplified schematic of the carotenoid biosynthesis pathway in DY59 strain from KEGG carotenoid pathway analysis.

Genes related to carotenoid biosynthesis in genus *Deinococcus*, which were linked to the phenotypic reddish color of the colonies, were detected *via* KEGG pathway analysis and genome-wide genomics studies (Tian and Hua, 2010; Lim et al., 2019). We selected four genes that are involved in the carotenoid biosynthesis pathway, which were marked in a simplified pathway schematic (Figure 2). The selected four carotenoid biosynthesis genes of strain DY59 exhibited amino acid sequence similarity with more than 60% to *D. geothermalis* genes: QR90_03795, a phytoene synthase, is 69.4% similar to Dgeo_0523; QR90_10400, a phytoene dehydrogenase (desaturase), is 78.9% similar to Dgeo_0524; QR90_14380, a carotenoid hydratase, is 58.6% similar to Dgeo_2309; and QR90_14400, a FAD-dependent oxidoreductase, is 74.5% similar to Dgeo_2306 (Supplementary Table S1). Interestingly, some carotenoid biosynthesis-related genes of *D. geothermalis* were adjacently clustered in the genome, for examples *crtB*-*crtI* and *cruC*-*cruD*-*cruF*-*crtO*. In contrast, each carotenoid biosynthesis gene in *D. radiopugnans* was separated.

### Distribution of IS elements in the DY59 genome

From the NCBI genome sequence data (submitted at December 2014) and protein information of DY59 (Kim et al., 2015), we first selected transposases and their genomic loci and defined full-length IS elements. The genome of strain DY59 contains six defined IS family members with DDE type transposase (Tpase) including IS*1*, IS*4*, IS*5*, IS*66*, IS*630*, and IS*701*, as well as five unclassified Tpases within a total of 36 ISs (Supplementary Table S2; Supplementary Figure S2). In general, Tpase is a major component of IS elements and simplified schematics of the IS structures except for the IS*1* family member, which has only a partial fragment, are illustrated in Supplementary Figure S3.

The IS*1* family member is an IS element (QR90_RS04110) with a 119 aa-length Tpase. In general, the IS*1* family IS element is composed of two transposase-related open reading frames (ORFs) with the same transcriptional direction. Therefore, TIR and DR sequences for this IS region of the IS*1* family could not be easily determined because it was only a partial fragment.

The IS*4* family members had five copies in the genome. The IS elements including transposases were separated into two subtypes. QR90_RS09690, QR90_04660, and QR90_01215 have identical IS elements with a 320 aa-long transposase, a conserved TIR sequence (CTCTGTACCGGACAACT), and DR sequences of variable sequences with 9 nt (“GCCGTGATC,” “GTCACGCCC,” and “GAAAGCAAT,” respectively). QR90_RS10860 and QR90_04150 possess another identical IS element with an identical transposase with 327 aa-length. Both IS elements have a conserved TIR sequence “CTCGGTAGCTGACAACTTCA” and DR sequences “AGATTGAAC” and “CAGGGTCAG.” Thus, these IS*4* family members were divided into the two subtypes IS*Drpg2* and IS*Drpg3*, with their Tpase sequences having a 67.19% amino acid identity.

The IS*5* family members have four copies in the genome with two subtypes elements: QR90_RS06350 with a 277 aa-length Tpase and QR90_RS04355, QR90_RS07350, and QR90_06585 with 265 aa-length Tpase sequences. The three 265 amino acid-long transposase genes are highly similar, with similarity rates of 97.7–100%. However, the Tpase of QR90_RS06350 with a 277 amino acid length exhibited a quite low similarity of 12–12.8% to the 265 aa-length Tpase in the IS*5* family. Therefore, the IS*5* family members were separated into two subtypes: IS*Drpg4* and IS*Drpg5*. The TIR and DR sequences of three IS elements with a 265 aa length Tpase were “AGGCTG” and “TAG,” respectively. The TIR and DR sequences of QR90_RS06350 were “ACCTCCTGCGAAAGTC” and “TAG,” respectively. The structural schemes for the forward and reverse area of the Tpase are identical, with a 54 and 9 nt distance, respectively. One interesting finding is that the loci of the IS*5* family IS elements are positioned close to the IS*701* family member, except for QR_ RS06350.

The IS*66* family IS elements have five copies in the DY59 genome: QR90_07275, QR90_RS09525, QR90_07340, QR90_11595, and QR90_09840, with a 472 aa-long Tpase named IS*Drpg6*. The IS elements have a conserved terminal inverted repeat (TIR) sequence “GTCTGTGATTAGCGGTCG” and 8 nt variable direct repeat (DR) sequences (“GATGGGGG,” “GGTGCAGG,” “ATGTCGTC,” “GGC GAGAG,” and “TATTTTTG”).

The IS*630* family members were divided into the two subtypes IS*Drpg7* and IS*Drpg8*: QR90_RS17010 with a 181 aa-long Tpase, QR90_RS08750, QR90_RS17180, QR90_RS17220, QR90_RS17305, and QR90_RS17410 with a 187 aa-long Tpase. Although QR90_17010 and QR90_RS17410 exhibited different Tpase lengths, the amino acid sequence similarities were 100% identical, whereas the 187 aa-length Tpases exhibited sequence identities ranging from 92.8 to 98.9%. In contrast, the Tpase of QR90_RS08750 exhibited a 64.7–67.9% identity when compared to the 187 aa-long Tpase. Thus, IS*Drpg7* included five IS elements except QR90_RS08750 for IS*Drpg8*. All of the examined IS*630* family members exhibited three-nucleotide DR sequences “TGA/TAA/TCA” and their TIR sequence was “TACGGACTCCGATTAA.”

The IS*701* family members included the 10 IS elements QR90_RS00720, QR90_04350, QR90_RS06590, QR90_05955, QR90_10425, QR90_RS17070, QR90_RS17080, QR90_RS17170, QR90_RS17235, and QR90_RS17395, with a 432 aa-length. Two distinct subtypes were identified according to Tpase identity: QR90_RS17070, QR90_RS17080, QR90_RS17170, QR90_RS17235, and QR90_RS17395 had a DNA sequence of 100% identity, whereas QR90_RS00720, QR90_04350, QR90_RS06590, QR90_05955, and QR90_10425 had a 99.5% identity. In contrast, the two subtypes exhibited only a 89.12% Tpase identity between each other. All IS*701* family members had a unique DR sequence (“nTAG”) and TIR sequence (“CTGTACTTTG GGGATATTCA”). Interestingly, the 3′ end of the TIR sequence of all IS*701* family members overlapped into the Tpase ORF.

In this study, we identified five unclassified IS members: QR90_08760 with a 270 aa-long Tpase; QR90_RS08625 with a 477 aa-long Tpase; QR90_08735 with a 434 aa-long Tpase; QR90_RS05950 with a 120 aa-long Tpase; and QR90_04880 with an 89 aa-long Tpase.

Next, the active transposition of IS elements was detected on non-pigment phenotypic selection under oxidative stress conditions induced by H_2_O_2_ treatment in the present study.

### Detection of active transposition on carotenoid biosynthesis by oxidative stress

A total of 24 and one non-pigmented colonies were detected after low concentration (80 and 100 mM) and high concentration (200 and 300 mM) H_2_O_2_ treatment for 1 h, respectively. It seems that generation of non-pigmented mutants is less efficient with higher H_2_O_2_ concentration.

**Figure 3:**
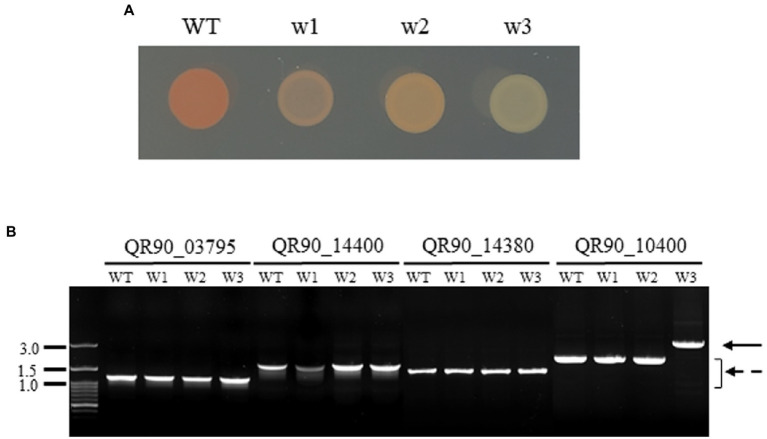
Phenotypic diversity in the high concentration of H_2_O_2_ treatment (200 mM for w1 and w2 or 300 mM for w3) for 1 h **(A)** and detection of gene disruption by PCR of genes involved in the carotenoid pathway **(B)**. QR90_03795, Phytoene synthesis; QR90_14400, FAD-dependent oxidoreductase; QR90_14380, carotenoid 1,2-hydratase; QR90_10400, phytoene dehydrogenase. Lanes: M, size marker; 1, 5, 9: WT; 2, 6, 10: w1; 3, 7, 11: w2; 4, 8, 12: w3. The dotted arrow indicates the wild-type gene PCR products. The solid arrow indicates the IS-integrated PCR product.

**Figure 4:**
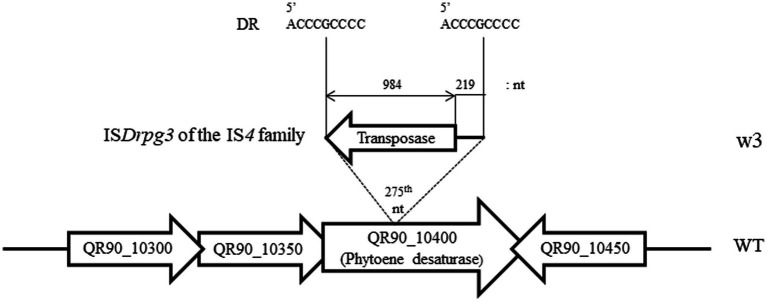
Detection of IS integration loci and composition of IS element in QR90_10400 phytoene desaturase in w3 mutant obtained after 300 mM H_2_O_2_ treatment. The IS*Drpg3* of the IS*4* family member was integrated at the 275th nt locus of the QR90_10400 gene with reverse transcriptional direction.

In the high-concentration treatment, one is a complete non-pigmented strain (w3) and two isolates exhibited a pale reddish color on TGY plates (w1 and w2; Figure 3A). When carotenoid biosynthesis genes were amplified by PCR with the appropriate primers, the complete non-pigmented strain only exhibited an enlarged PCR product from QR90_10400, a phytoene desaturase (Figure 3B). However, other carotenoid biosynthesis genes were not affected. The IS*Drpg3* of the IS*4* family was integrated at the 275th nucleotide of QR90_10400 (Figure 4). The TIR sequence of this IS element was “CTCGGTAGCTGACAACTTCA” and the DR sequence was “ACCCGCCCC.”

**Figure 5:**
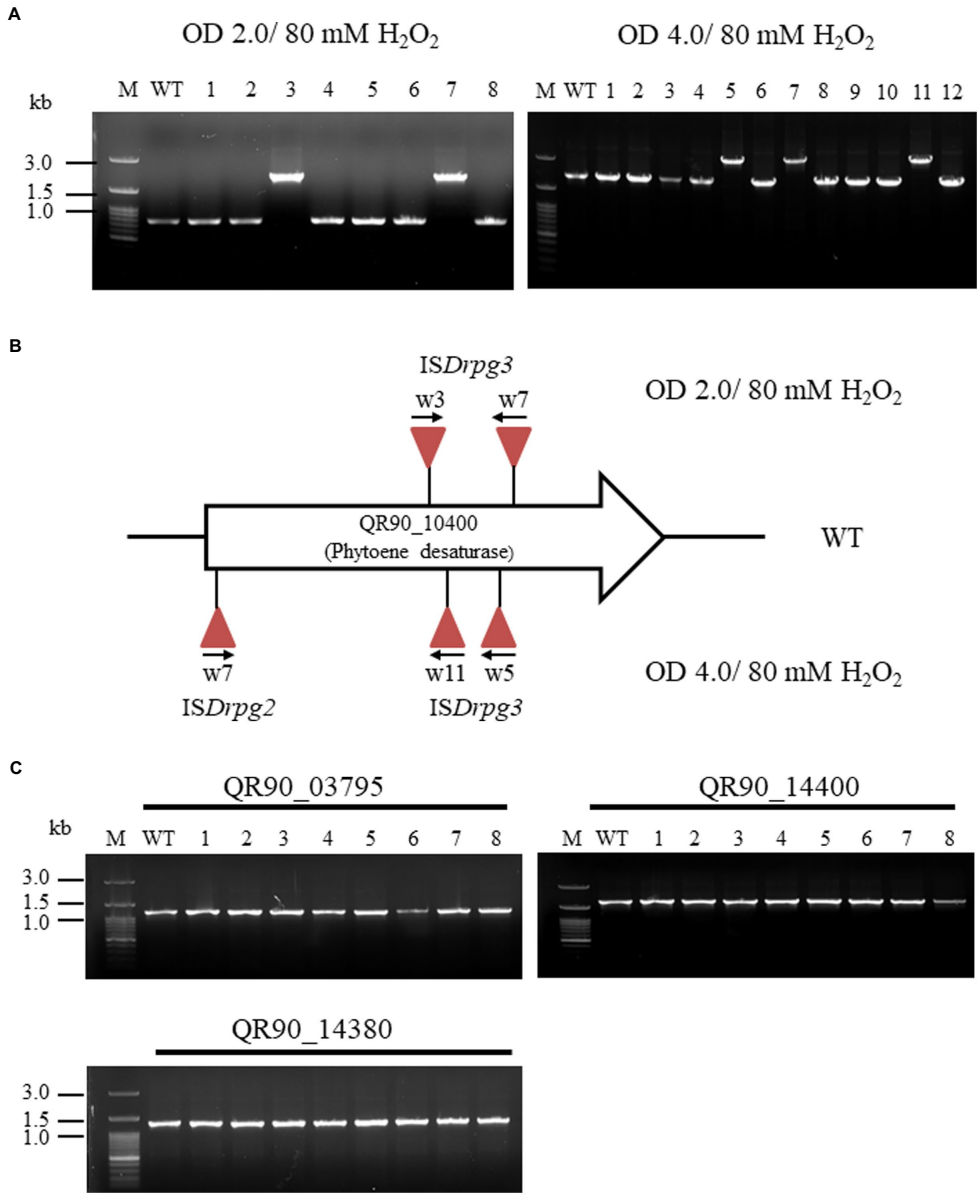
Insertion sequence transposition triggering at low concentration of H_2_O_2_ treatment with 80 mM. **(A)**, Detection of IS transposition at two growth phases (OD_600_ 2.0 and 4.0) in gene QR90_10400. **(B)**, Analysis of IS integrational loci at both detection conditions. All transposed IS elements are both IS*Drpg2* and IS*Drpg3* of the IS*4* family members. **(C)**, Analysis of IS transposition in three carotenoid biosynthesis-related genes. These genes were not affected by IS integration under H_2_O_2_ oxidative stress conditions.

In the low-concentration H_2_O_2_ treatment, five out of 24 non-pigmented strains exhibited PCR amplicons with QR90_10400 gene enlargement: two strains with an OD_600_ of 2.0 and three strains with an OD_600_ of 4.0 at 80 mM H_2_O_2_ (Figure 5A). The frequency of IS transposition in gene QR90_10400 exhibited 5.1 × 10^−4^ and 1.8 × 10^−4^ from OD_600_ of 2.0 and 4.0, respectively (Supplementary Table S4). All five of these IS-integrated mutants exhibited IS*4* family transposition (Figure 5B; Supplementary Figure S4). Other carotenoid-related genes QR90_03795, QR90_14380, and QR90_14400 were not affected (Figure 5C). The 19 remaining clones did not exhibit any changes in the size of the PCR products, indicating that there was no IS transposition in the selected four carotenoid biosynthesis genes. However, there might be point mutations in carotenoid-related genes or perhaps IS transposition in non-analyzed carotenoid biosynthesis-related genes.

At an OD_600_ of 2.0, one IS element (w3) was integrated in the forward direction at the 401st nt of phytoene desaturase. The DR sequence of this element was “CTTCTTCGA” and the TIR sequence was “CTCGGTAGCTGACAACTTCA.” Another (w7) integrated in the reverse direction at the 617th nt. The DR sequence of this element was “GTAAACGAG” and the TIR sequence was “CTCGGTA GCTGACAACTTCA” (Figure 5B; Supplementary Figure S4A). Both IS elements were belonged to IS*Drpg3* of QR90_04150 or QR90_RS10860 (Supplementary Table S2).

At an OD_600_ of 4.0, one IS element (w5) integrated in the reverse direction at the 587th nt of phytoene desaturase. The DR sequence of this element was “AGGCGCTC” and the TIR sequence was “CTCGGTAGCTGACAACTTCA.” Another (w11) integrated in the reverse direction at the 474th nt; its DR sequence was “GCTCGTAGC” and its TIR sequence was identical to that of w5. Both IS elements were identical to IS*Drpg3* (Supplementary Figure S4B). The last IS element (w7) integrated in the forward direction at the 22nd nt with DR sequence “CCAGCAGGC” and TIR sequence “CTCT GTACCGGACAACT” (Figure 5B; Supplementary Figure S4C). This IS element was identical to IS*Drpg2* of QR90_01215, QR90_04660, or QR90_09690. Therefore, *D. radiopugnans* DY59 exhibits active transposition of IS*4* family members in H_2_O_2_ treatment conditions. The PCR detection of five copies of IS*4* family members at the location as found in the genome sequence indicates that the active transposition occurred through replicative mode in present (Supplementary Figure S5).

### Effects of hypochlorite and gamma-irradiation in IS transposition

The 100 μM sodium hypochlorite does not affected viability of the strain DY59. 5 kGy irradiated strain DY59 exhibits 99.5% reduction of CFU. The selection of non-pigmented colonies was performed *via* gamma irradiation exposure and sodium hypochlorite treatment. Unlike the lack of pigment production of *D. geothermalis*, gamma irradiation of total 5 kGy did not induce non-pigment phenotypes in the *D. radiopugnans* DY59 wild-type strain (Ye et al., 2022). Moreover, although hypochlorite treatment induced the non-pigment phenotype, the non-pigmented colony did not exhibit IS element integration in the four analyzed carotenoid biosynthesis genes (data not shown). This may be the same explanation for the absence of transposition of IS in phenotypic changes due to point mutations in four carotenoid biosynthetic genes and the decay of other genes related to pigment formation.

### Expression levels of catalase, three LysR family regulators included oxyR and Tpase of the IS4 family by qRT-PCR analysis

**Figure 6:**
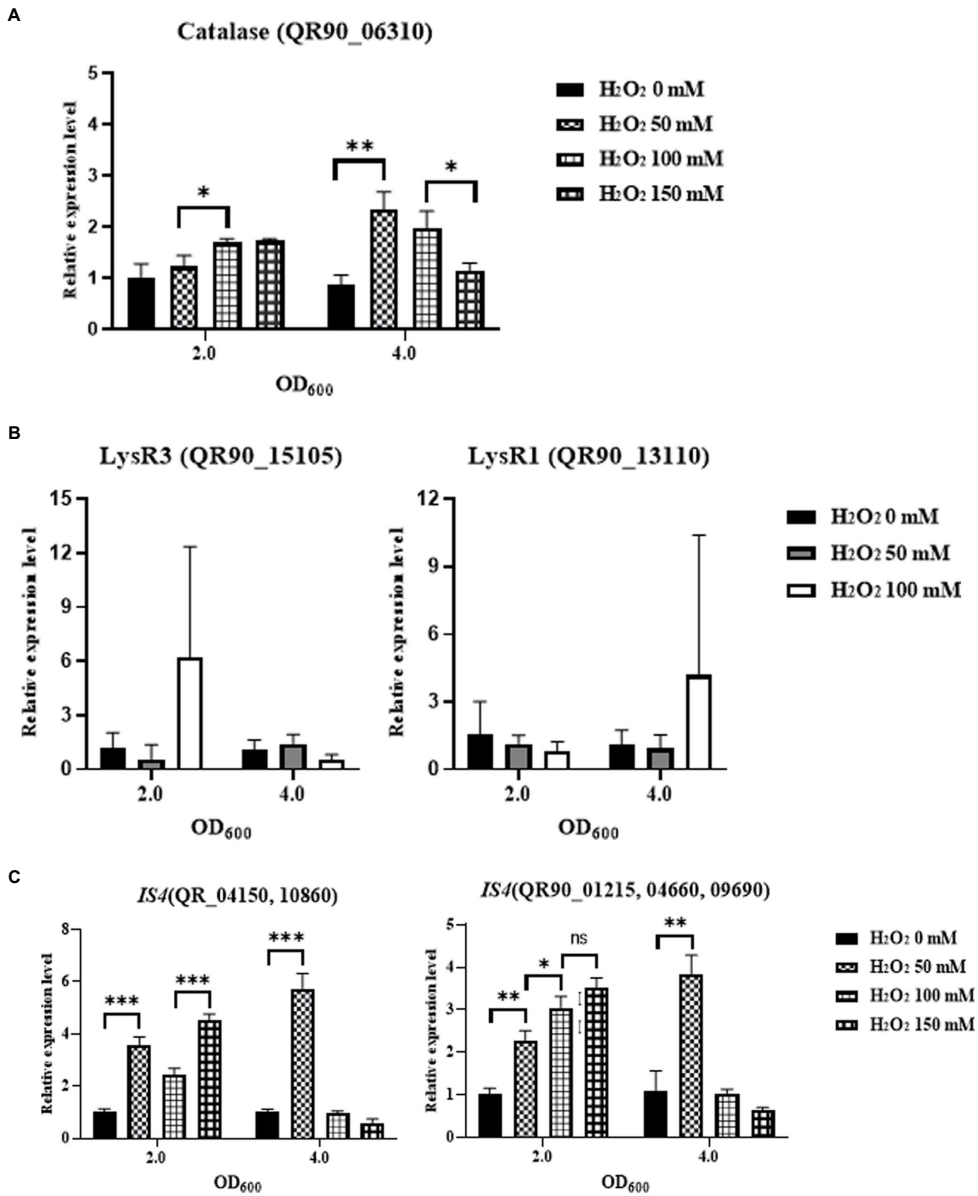
Comparison of the gene expression levels of catalase **(A)**, two LysR family regulators **(B)** and two transposases in IS*Drpg2* and IS*Drpg3* **(C)** by qRT-PCR under different final concentrations of H_2_O_2_ (0–150 mM). Pair-wise comparisons between experimental groups were conducted *via* Student’s *t*-test with the Prism™ ver. 8.0 software (^*^p < 0.05; ^**^p < 0.01; ^***^p < 0.001).

To determine the mechanisms underlying the high H_2_O_2_ resistance of DY59, the expression levels of catalase and three LysR family regulators including possible *oxyR* were first measured by qRT-PCR analysis. The chromosome of DY59 strain has a single catalase QR90_06310 with 72.17 and 74.17% amino acid sequence similarity to *D. radiodurans* catalase KatE1 and *D. geothermalis* KatE, respectively. After exposure to various concentrations of H_2_O_2_ (50, 100, 150 mM, and an unexposed control) for 1 h and growing the cells to OD_600_ values of 2.0 and 4.0, the relative expression levels of catalase and LysR family members were measured *via* qRT-PCR by the basal expression level of OD_600_ of 2.0 with unexposed control as 1.0. Unexpectedly, catalase was not dramatically induced at OD_600_ 2.0 or 4.0 in any of the tested H_2_O_2_ concentrations. Nevertheless, there was a 2-fold increase in catalase expression at OD_600_ 4.0 when the cells were challenged with 50 and 100 mM H_2_O_2_ (Figure 6A).

LysR1 QR90_13110 exhibited a more than 6-fold induction at an OD_600_ of 4.0 upon exposure to 100 mM H_2_O_2_ and LysR3 QR90_15105 exhibited a more than 6-fold induction at an OD_600_ of 2.0 in the 100 mM H_2_O_2_ condition. Our findings thus indicated that 100 mM H_2_O_2_ controlled the expression of LysR1 and LysR3 at two different growth phases (Figure 6B). However, LysR2 (QR90_14595) was not affected regardless of growth phase and oxidation condition (data not shown). LysR2 was exhibited 70.3 and 75.7% identities of amino acid sequence to the proposed OxyR of *D. radiodurans* and *D. geothermalis*, respectively. Therefore, the high H_2_O_2_ resistance phenotype of the DY59 strain cannot be attributed to changes in the expression of catalase and its three potential LysR family regulator members, as determined by RNA production levels measured by qRT-PCR analysis.

There are two subtypes of IS*4* family which were actively transposed to other loci. Tpase of IS*Drpg3* such as QR90_04150 and QR90_10860/IS*Drpg2* such as QR90_01215, QR90_04660, and QR90_09690 exhibited a more than 5-6-fold induction at an OD_600_ of 4.0 in the 50 mM H_2_O_2_ condition. The related expression levels were reduced at an OD_600_ of 4.0 in the 100 and 150 mM H_2_O_2_ condition (Figure 6C). However, both IS*Drpg2* and IS*Drpg3* Tpases were gradually induced over 2-fold at an OD_600_ of 2.0 in the different H_2_O_2_ conditions. Thus, IS elements of the IS*4* family were induced by the H_2_O_2_ and actively transposed into other genomic loci with replicated mode.

## Discussion

*Bacillus subtilis*, a widely known Gram-positive bacterial model organism, lacks IS or any other transposable element excluding several bacteriophages and other remnants of horizontal gene transfer (HGT) events from genome study of wild-type strain. The IS identification platform “ISfinder” revealed four IS families in the *B. subtilis* genome including IS4*Bsu1* (IS*4* family), IS*Bsu1* (IS*3* family), IS*Bsu2* (IS*256* family), and IS*Bsu3* (IS*1595* family; Siguier et al., 2006). Interestingly, pathogenic *Bacillus* species such as *B. cereus*, *B. anthracis*, *B. thuringiensis,* and others have many IS families and Tn3 elements (Fayad et al., 2019). Therefore, the distribution of transposable elements may be associated with pathogenicity. Additionally, the genomes of the pathogenic bacteria may have undergone multiple acquisition of transposable elements through HGT in response to environmental stimuli (Fayad et al., 2019).

Here, we detected active transposition of IS elements under oxidative stress conditions of H_2_O_2_ treatment in the radiation-resistant bacterium *D. radiopugnans* DY59, which was isolated from mountain soil collected in South Korea. The DY59 isolate was phylogenetically clustered near three *Deinococcus* species from Antarctic marine environments (Hirsch et al., 2004; Kim et al., 2015). The genome of *D. radiopugnans* DY59 has a total of 36 IS elements encompassing six IS families. Interestingly, these six family members belonged to *D. geothermalis* IS families that also contained the IS*6*, IS*982*, and IS*200/605* families (Lee et al., 2020). Unfortunately, the IS names cannot be classified based on the IS distribution in the *D. radiopugnans* genome from “ISfinder” platform (at Sep. 2022; Siguier et al., 2006). The transposase annotation as a key component of IS element was varied among the genome data and between prediction and analysis.

When wild-type DY59 cells were treated with 80 or 300 mM H_2_O_2_, the active transposition of the IS*4* family members was only detected on phytoene desaturase (QR90_10400; Figures 4, 5). The active transposition of the IS*4* family was also particularly detected in wild-type *D. geothermalis* upon H_2_O_2_ treatment and dielectric bilayer discharge (DBD) plasma radiation (Lee et al., 2020; Ye et al., 2022).

When the *D. radiodurans* strains were exposed to gamma irradiation, there were two particular active transposition events on a trimethoprim-resistant selection. One was an IS integrated into a *thyA* gene; the other was an *uvrA* gene disruption that resulted in mitomycin-resistant phenotypic selection (Narumi et al., 1997; Mennecier et al., 2006; Pasternak et al., 2010). When the cells were treated with 5 kGy of gamma irradiation, the wild-type *D. geothermalis* strain exhibited active transposition of IS families, (e.g., IS*1* and IS*5* family), whereas the wild-type DY59 strain and *D. radiodurans* strain did not exhibit non-pigment phenotypic mutations (Ye et al., 2022).

Insertion sequence transposition has been detected under various environmental stressors, such as nutrient deprivation, temperature changes, metal ion exposure, and oxidative stress caused by UV irradiation, gamma irradiation, and H_2_O_2_ treatment (Ohtsubo et al., 2005; Twiss et al., 2005; Kharat et al., 2006; Mijnendonckx et al., 2011; Suzuki et al., 2021). Interestingly, this active transposition of IS elements was found to vary in a species-specific manner. *D. geothermalis* wild-type and several particular gene-disrupted mutants have been reported to exhibit different types of IS transposition. For example, IS*Dge3* of the IS*1* family was actively transposed under gamma irradiation, whereas IS*Dge11* of the IS*4* family was transposed under H_2_O_2_ treatment and DBD plasma radiation. Moreover, IS*Dge5* and IS*Dge6* of the IS*5* family were found to be transposed in *dps*-, *oxyR*-, cystine importer-, and *lysR*-deficient mutants (Lee et al., 2020, 2022; Ye et al., 2021).

Here, we explored the IS distribution in the genome size of 3.54 Mb of the DY59 strain. Our findings suggested that DY59 exhibits a less complex IS distribution than that of *D. geothermalis*. In this study, a strict IS element of the IS*4* family was actively transposed into a carotenoid biosynthesis gene QR90_10400 phytoene desaturase under H_2_O_2_ treatment (Figures 4, 5B; Supplementary Figure S4). Therefore, the wild-type *D. radiopugnans* DY59 may serve as a suitable model organism for studying the active transposition of unique IS element using a single oxidation inducer such as H_2_O_2_. These experiments provide the opportunity to determine unique IS transposition events in organisms that exhibit genomic plasticity. However, there are still many challenges associated with IS naming and classification from gene annotation, emphasizing the need for more accurate algorithms and additional criteria including machine learning tools for IS identification and assignment, as well as the creation of a global network of research groups working together.

The genome sequence information of *D. radiopugnans* ATCC19172 was updated twice on June 2019 and August 2020, with contig assembly lengths of 4.33 and 4.3 Mb, respectively (NCBI genomes). *Deinococcus radiopugnans* ATCC19172 has three catalases, FHR04_11220, FHR04_17100, and FHR04_17320, sharing, respectively, 99, 26.6, and 47.6% identity with the single identified catalase (QR90_06310) from strain DY59. In case the published genome sequence of DY59 is incomplete, this strain might also possess homologs of FHR04_17100 and FHR04_17320. Thus, we performed qRT-PCR using primer sets corresponding to FHR04_17100 and FHR04_17320 to investigate expression of possible homologs in strain DY59. The possible FHR04_17100 homolog was not induced by the H_2_O_2_ treatment and the possible FHR04_17320 homolog exhibited more than 2-fold induction at OD_600_ of 2.0 with 50 and 100 mM H_2_O_2_ treatment (Supplementary Figure S6). When 150 mM H_2_O_2_ treatment at OD_600_ of 4.0 was applied, the possible FHR04_17320 homolog exhibited nearby 3-fold induction. In the present additional catalase expression data, the high hydrogen peroxide resistance of DY59 is not explained by induced expression of catalase genes. DY59 strain may have a high level of constitutive catalase enzyme activity, or it may employ other mechanisms for hydrogen peroxide resistance, such as unidentified protective pathways and physiological defense systems using extracellular matrixe components such as extracellular polymeric substances (EPS), proteins, and eDNA, which aid in biofilm formation and enhance tolerance to oxidative stress, and certain transporters (Li et al., 2013; Molina-Santiago et al., 2021).

The genome sequence of strain ATCC19172 is 0.8 Mb larger than that of DY59. Although the reported genome sequence of strain DY59 consist of only a chromosome, we consider the possibility that this strain might have one or more additional genome molecules such as plasmids. If the strain DY59 has plasmids, the IS family and number will expand. IS elements are commonly known to exhibit random movement. However, experiments conducted on *Deinococcus geothermalis* have shown that the transposition of unique IS elements follows a specific pattern. The exact pattern is still unknown, but this experiment provides a starting point for further research. Specifically, it was observed that the IS*4* family of IS element only transposed under hydrogen peroxide conditions in *D. radiopugnans* DY59. Additionally, the ability of *Deinococcus* species to endure various forms of stress such as radiation, drying, and toxic chemicals is attempted to be explained from a molecular evolutionary perspective through the transposition of IS elements. Further exploration into this research area presents a significant challenge for the future.

## Data availability statement

The datasets presented in this study can be found in online repositories. The names of the repository/repositories and accession number (s) can be found in the article/Supplementary material.

## Author contributions

ES, HN, QY, and S-JL performed conception, designed experiments, and performed and analyzed data. ES, HN, and S-JL wrote the manuscript. All authors contributed to the article and approved the submitted version.

## Funding

This study was supported by the National Research Foundation of Korea (NRF) grant funded by the Korean government (MS&ICT; 2022R1A2C1010233).

## Conflict of interest

The authors declare that the research was conducted in the absence of any commercial or financial relationships that could be construed as a potential conflict of interest.

## Publisher’s note

All claims expressed in this article are solely those of the authors and do not necessarily represent those of their affiliated organizations, or those of the publisher, the editors and the reviewers. Any product that may be evaluated in this article, or claim that may be made by its manufacturer, is not guaranteed or endorsed by the publisher.
