# Supplementary figures and images for: Hydrogen peroxide treatment induces the transposition of an insertion sequence in Deinococcus radiopugnans DY59
Source: Front Microbiol. 2023 Mar 2;14:1110084. doi: 10.3389/fmicb.2023.1110084 (PMC10017437; doi:10.3389/fmicb.2023.1110084)

Fig. S1

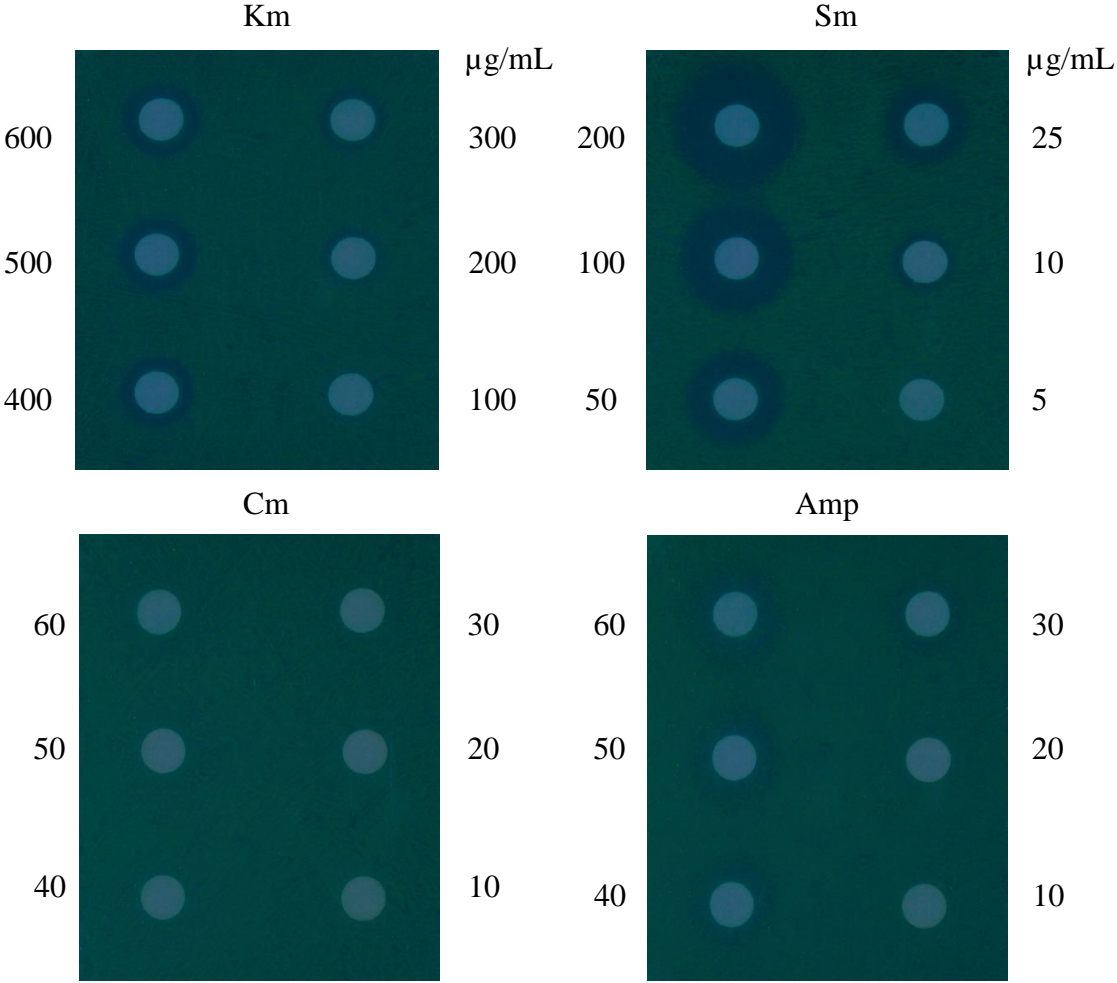

Fig. S2

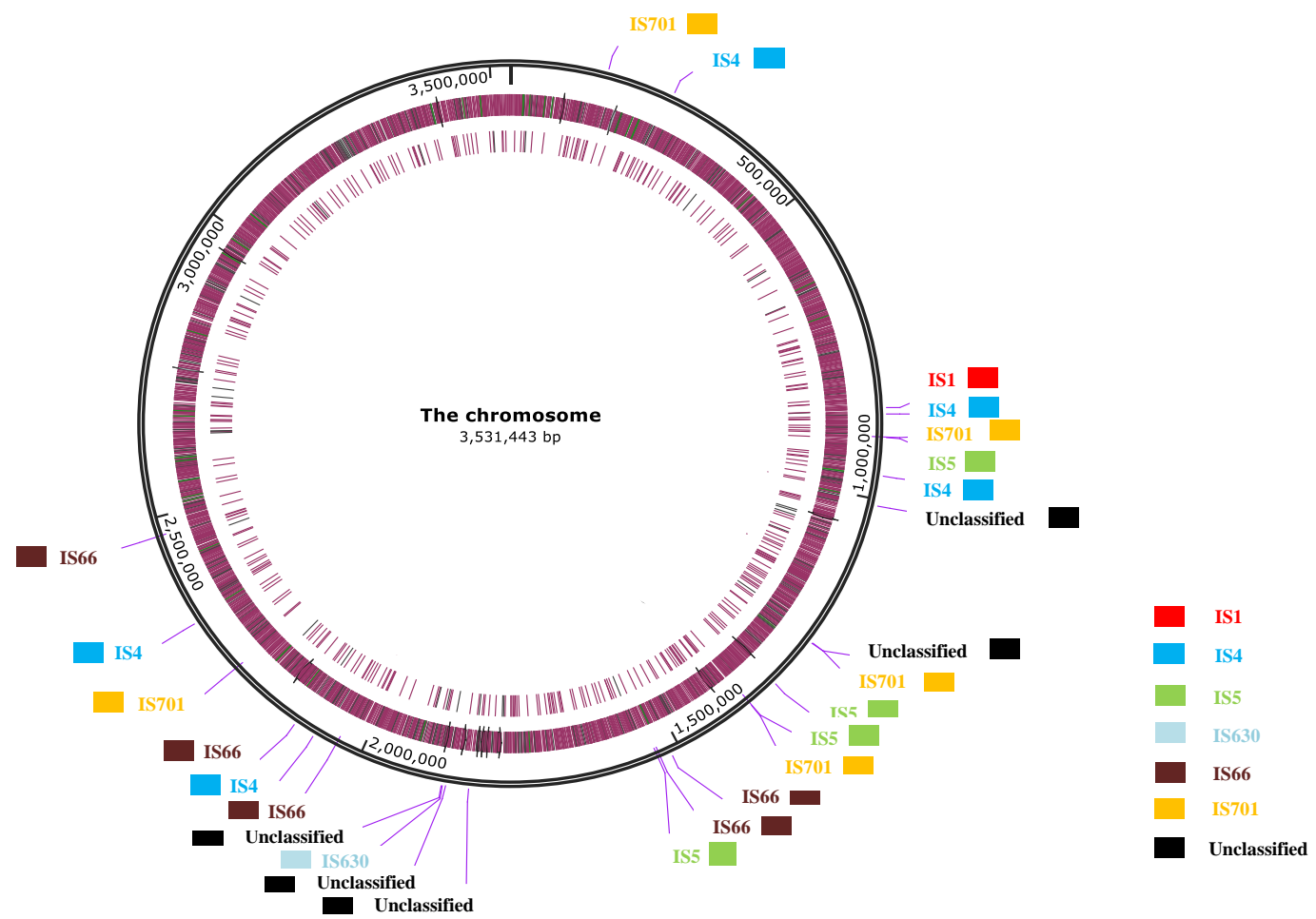

Fig. S3

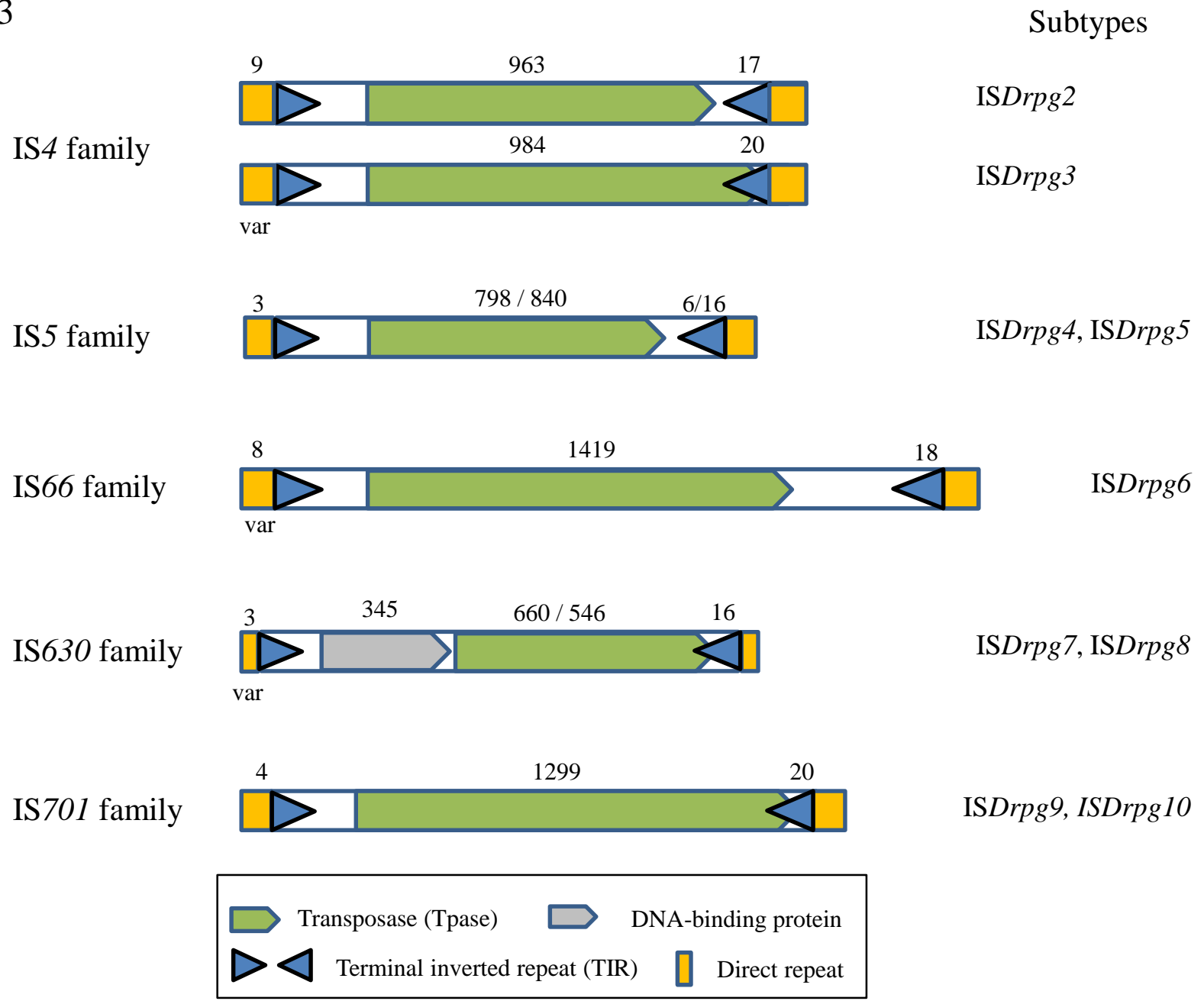

Fig. S4

A

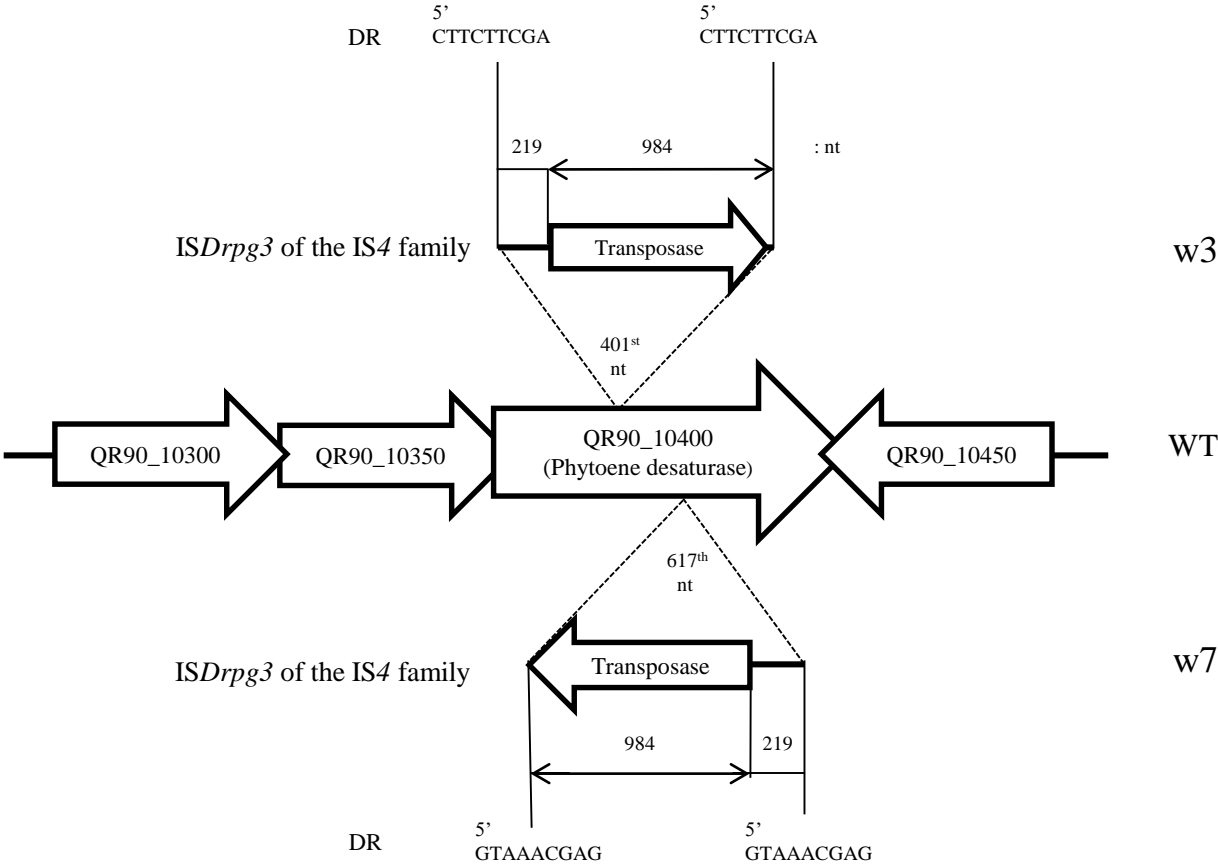

Fig. S4

B

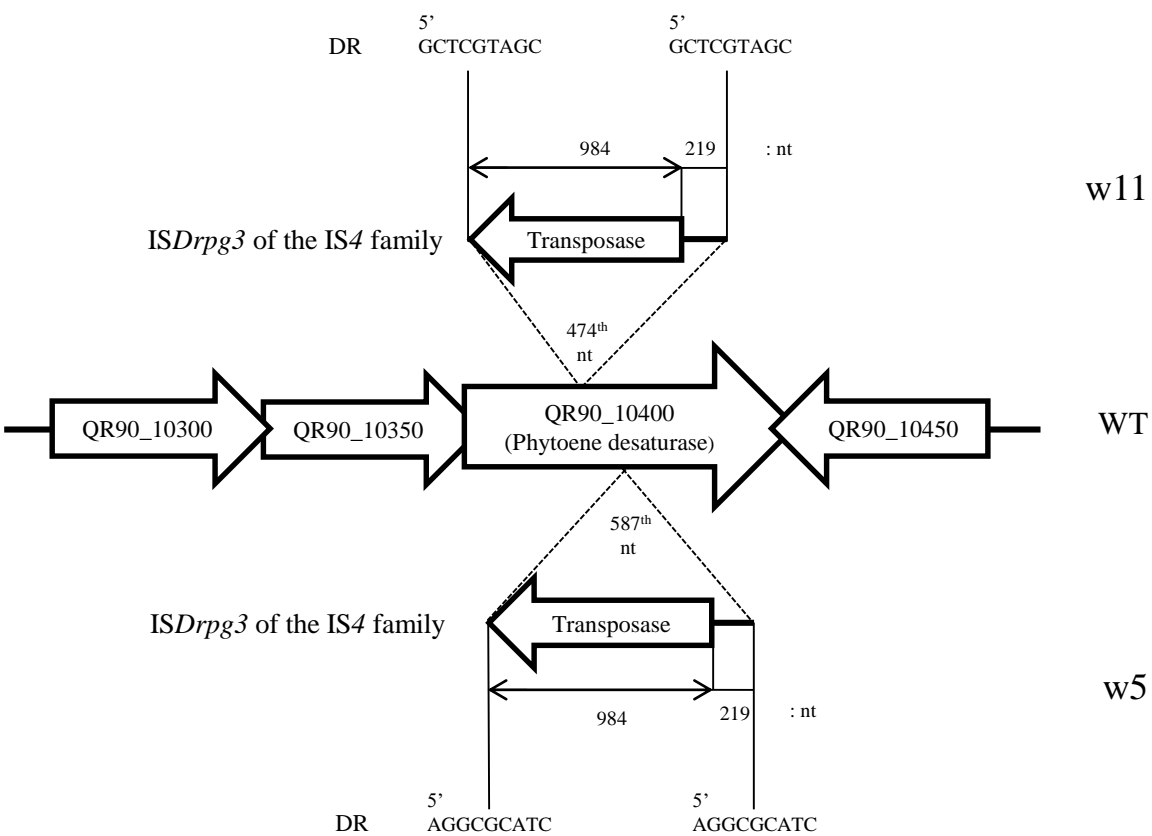

Fig. S4

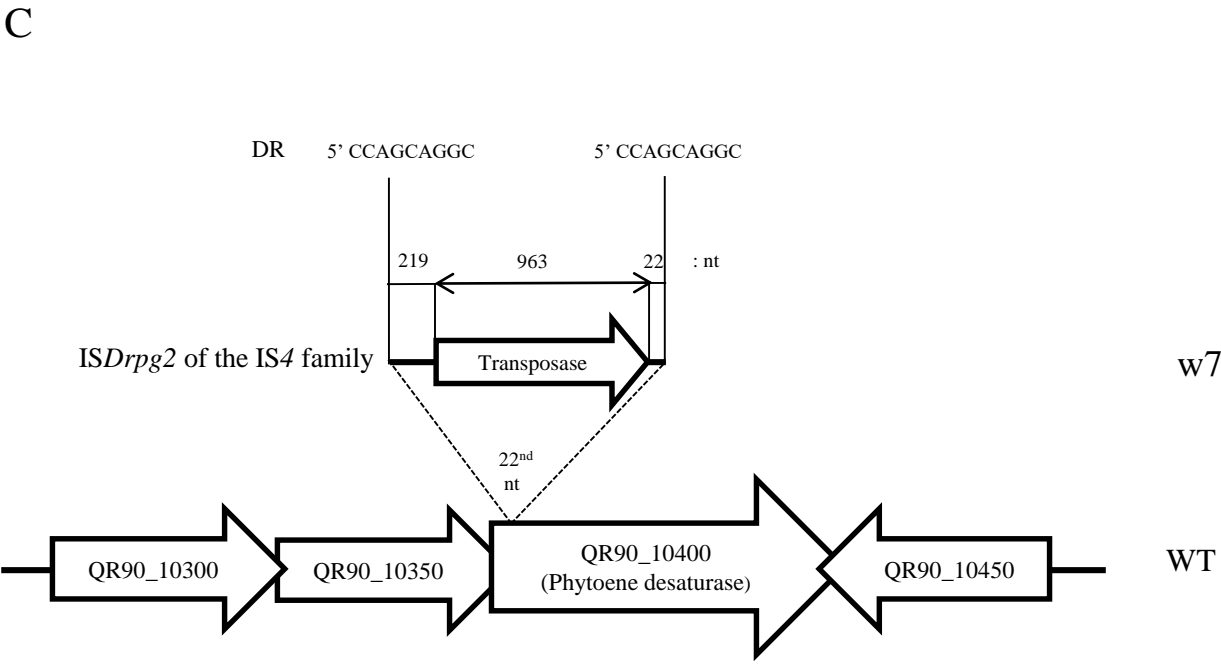

Fig. S5

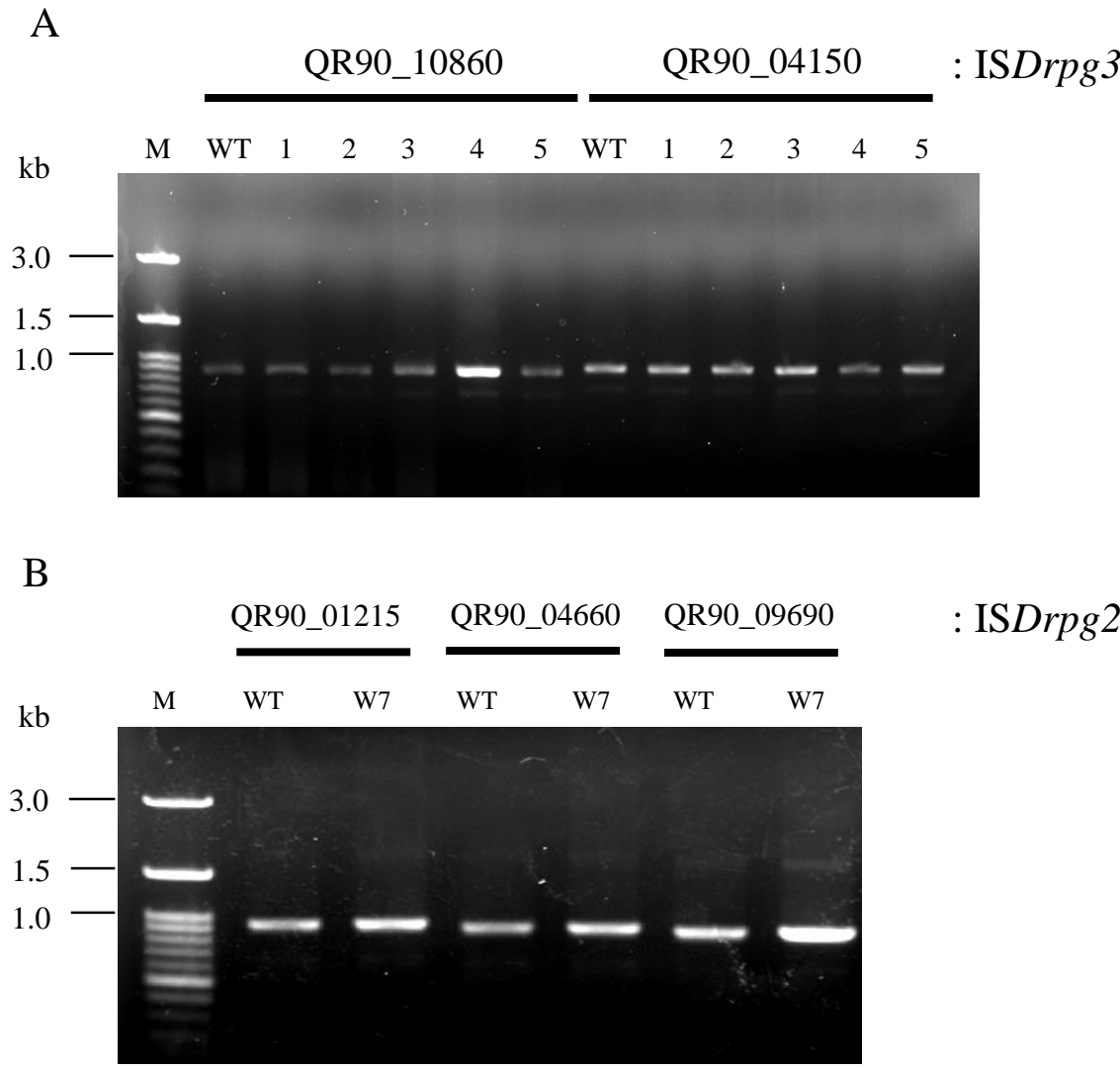

Fig. S6

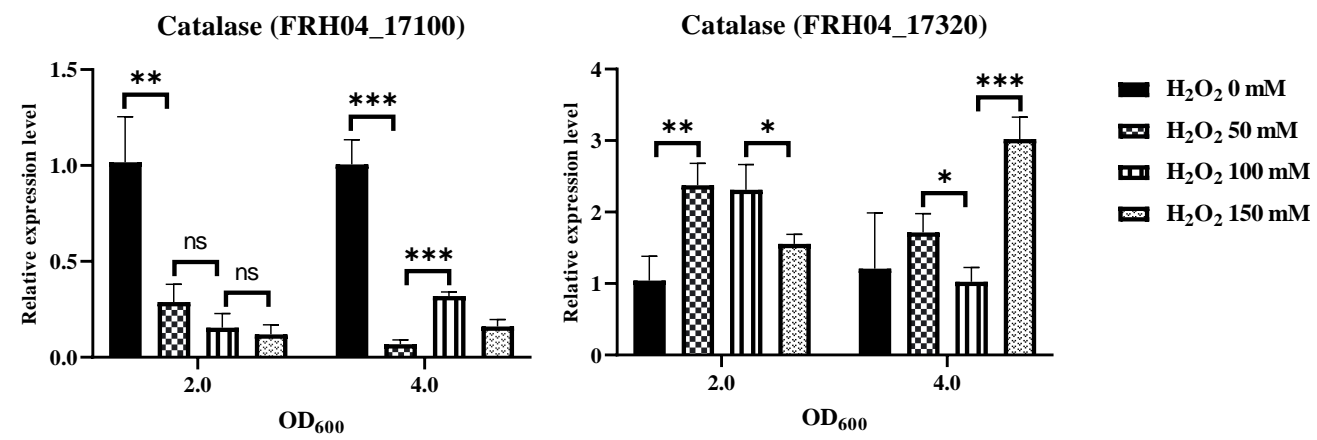

Supplement: Supplementary file 1 [file Data_Sheet_1.pdf]
