# Supplementary material for: Hydrogen peroxide treatment induces the transposition of an insertion sequence in Deinococcus radiopugnans DY59
Source: Front Microbiol. 2023 Mar 2;14:1110084. doi: 10.3389/fmicb.2023.1110084 (PMC10017437; doi:10.3389/fmicb.2023.1110084)
Supplement: Supplementary file 2 [file Data_Sheet_2.pdf]

Supplementary data

Table S1. Genetic arrangement of carotenoid biosynthesis-related genes between DY59 and *D. geothermalis* genomes.

D. radiopugnans DY59		*D. geothermalis*		Sequence similalrity	
Gene	products	Gene	Products	Nucleotide	Amino acid
QR90_03795	Phytoene synthase	Dgeo_0523	Phytoene synthase, CrtB	67.5%	69.4%
QR90_10400	Phytoene dehydrogenase	Dgeo_0524	CrtI, Phytoene dehydrogenase	74.1%	78.9%
QR90_14380	Carotenoid 1,2-hydratase	Dgeo_2309	Carotenoid biosynthesis protein	58.6%	58.6%
QR90_14400	FAD-dependent oxidoreductase	Dgeo_2306	FAD-dependent oxidoreductase	71.5%	74.5%

Table S2. Distribution of IS elements in *D. radiopugnans* DY59 genome

Types	IS family	Tpase (aa)	Loci of Tpase	Note
IS*Drpg1*	IS*1*	QR90_04110 (106 aa)	857791.. 858150	partial
IS*Drpg2*	IS*4*	QR90_01215 (320 aa)<br>QR90_04660 (320 aa)<br>QR90_RS09690 (320 aa)	254157.. 255119<br>964528.. 965490<br>2076363.. 2077325	TIR : CTCTGTACCGGACAACT<br>DR : variable 9 nt
IS*Dprg3*	IS*4*	QR90_04150 (327 aa)<br>QR90_RS10860 (327 aa)	867329..868312<br>2324778..2325761	TIR : CTCGGTAGCTGACAACTTCA<br>DR : variable 9 nt
IS*Dprg4*	IS*5*	QR90_RS04355 (265 aa)<br>QR90_06585 (265 aa)<br>QR90_RS07350 (265 aa)	904878..905675<br>1373354..1374151<br>1536028..1536825	TIR : AGGCTG<br>DR : TAG
IS*Dprg5*	IS*5*	QR90_RS06350 (278 aa)	1325693..1326529	TIR : ACCTCCTGCGAAAGTC<br>DR : TAG
IS*Dprg6*	IS*66*	QR90_RS07275 (469 aa)<br>QR90_RS07340 (469 aa)<br>QR90_RS09525 (469 aa)<br>QR90_RS09840 (469 aa)<br>QR90_RS11595 (469 aa)	1514842.. 1516251<br>1530399.. 1531808<br>2038906.. 2040315<br>2109905.. 2111314<br>2468275.. 2469684	TIR : GTCTGTGATTAGCGGTCG<br>DR : variable 8 nt
IS*Dprg7*	IS*630*	QR90_RS17010 (181 aa)<br>QR90_RS17180 (187 aa)<br>QR90_RS17220 (187 aa)<br>QR90_RS17305 (187 aa)<br>QR90_RS17410 (187 aa)	41158.. 41649<br>1683287.. 1683796<br>2012618.. 2013127<br>2458990.. 2459499<br>3296144.. 3296653	TIR : TACGGACTCCGATTAA<br>DR : variable 3 nt
IS*Dprg8*	IS*630*	QR90_RS08750 (187 aa)	1867932.. 1868495	TIR : TACGGACTCCGATTAA<br>DR : TGA
IS*Dprg9*	IS*701*	QR90_RS00720 (432 aa)<br>QR90_RS04350 (432 aa)<br>QR90_RS05955 (432 aa)<br>QR90_RS06590 (432 aa)<br>QR90_RS10425 (432 aa)	149514.. 150812<br>903544.. 904842<br>1242762.. 1244060<br>1374188.. 1375486<br>2231829.. 2233127	TIR : CTGTACTTTGGGGATATTCA<br>DR : nTAG
IS*Dprg10*	IS*701*	QR90_RS17070 (432 aa)<br>QR90_RS17080 (432 aa)<br>QR90_RS17170 (432 aa)<br>QR90_RS17235 (432 aa)<br>QR90_RS17395 (432 aa)	905757..907055<br>994882.. 996180<br>1536917.. 1538215<br>2074796.. 2076094<br>3244761.. 3246059	TIR : CTGTACTTTGGGGATATTCA<br>DR : nTAG
	Unclas-sified	QR90_08760 (270 aa)<br>QR90_RS08625 (477 aa)<br>QR90_08735 (434aa)<br>QR90_RS05950 (120 aa)<br>QR90_04880 (89 aa)	1869233..1870045<br>1828606.. 1830039<br>1863843.. 1865147<br>1241759.. 1242121<br>1009933.. 1010202	

Table S3. List of qRT-PCR primers in this study.

qRT-PCR primer	Product	Sequence
QR90_07845_F	glyceraldehyde-3-phosphate dehydrogenase	CATGCTGTCCTCGTCGTAGT
QR90_07845_R	glyceraldehyde-3-phosphate dehydrogenase	GCGATCAATGACCTGACCG
QR90_13110_F	LysR family transcriptional regulator	GCCGTCACGCTGAACATC
QR90_13110_R	LysR family transcriptional regulator	ATCAGCGCGAGATCCGTCT
QR90_14595_F	LysR family transcriptional regulator	TTGATGCTGCTCCCCGTT
QR90_14595_R	LysR family transcriptional regulator	AAATCTTGAGCCGGCGCTT
QR90_15105_F	LysR family transcriptional regulator	TCAGAACCAGCCCACCTT
QR90_15105_R	LysR family transcriptional regulator	CGACTGCGAGGCATTCAACT
QR90_04150, 10860_F	Transposase (IS4-1)	CGGCACCAGTTCTCCTGAA
QR90_04150, 10860_R	Transposase (IS4-1)	CAGAAAGACCTGAGCCGTGA
QR90_01215, 04660, 09690_F	Transposase (IS4-2)	ACTTGGGAACGTGGGGAATC
QR90_01215, 04660, 09690_R	Transposase (IS4-2)	TGTTCCCATCGTGATCGAGG
QR90_06310_F	catalase	AAGGTGTTCTTCATCCGCGA
QR90_06310_R	catalase	CATGAAGTCGAAGATGCGCC
FHR04_17100 F	catalase	ACAAGCTGCAGTACACCGTC
FHR04_17100 R	catalase	GAACAGGTATTGCAGGCACAC
FHR04_17320 F	Catalase	ATTCACAGCCAGAAACGCCA
FHR04_17320 R	catalase	GGTCCCCGAACATGTAGACC

Table S4. Colony forming units and frequency of IS transposition in different oxidative stress conditions.

OD/H_2O_2 Conc.	*CFU	**IS transposition	Frequency of active transposition
2.0/80 mM	$1.97x10^8$	$1x10^5$	$5.1 x 10^{-4}$
4.0/80 mM	$8.3x10^8$	$1.5x10^5$	$1.8 x 10^{-4}$
4.0/100 mM	$6.12x10^8$	-	-

*CFU/ml for the culture after 1 h H_2O_2 treatment

**ratio of IS transposition events in culture of 1 ml
